# Complete chloroplast genome sequence and annotation of *Machilus salicina* Hance, 1885 (lauraceae)

**DOI:** 10.1080/23802359.2022.2068981

**Published:** 2022-05-04

**Authors:** Long Xiaoxuan, Wenbo Shi, Weicai Song, Weiqi Han, Guiwen Yang, Shuo Wang

**Affiliations:** a College of Marine Science and Biological Engineering, Qingdao University of Science and Technology, Qingdao, China; b Shandong Provincial Key Laboratory of Animal Resistance Biology, College of Life Sciences, Shandong Normal University, Jinan, Shandong, China

**Keywords:** *Machilus salicina*, Lauraceae, chloroplast genome, phylogenetic relationships

## Abstract

*Machilus salicina* Hance, 1885 is a species of flowering plant in the family Lauraceae and is mainly found at low altitudes in southern China. In this study, we assembled and annotated the complete chloroplast genome of *M. salicina* for the first time. We analyzed the general features of *M. salicina* and constructed a phylogenetic tree based on 15 Lauraceae species. The chloroplast genome of *M. salicina* had a total length of 153,943 bp. The length of a large single copy region, a small single copy region, and two inverted repeat regions were 93,689 bp, 20,070 bp, and 20,092bp, respectively. A total of 128 genes were detected, which included 84 protein-coding genes, 36 tRNAs and 8 rRNAs. The GC content of *M. salicina* complete chloroplast genome was 39.1%. The phylogenetic tree indicated that *M. salicina* was closely related to *M. yunnanensis*.

*Machilus salicina* Hance, 1885, is a species of flowering plant in the family Lauraceae and is mainly found at low altitudes in Guangdong, Guizhou, and Yunnan provinces of China. The leaves of *M. salicina* are dense and can be used as herbs to help reduce swelling and detoxify the bod*y* (Zhijun and Young 2003; Zuo et al. 2012; Chen et al. 2020). As the cost of next-generation sequencing technology has decreased, chloroplast genomes have increasingly been sequenced (Ge et al. 2019; Song et al. 2022). The chloroplast genomes of *Machilus* (*M. yunnanensis*, *M. balansae*, and *M. robusta*) have been reported in previous studies (Song et al. 2015; Wu et al. 2021). However, the chloroplast genome of *M. salicina* has yet to be reported. In this study, we assembled and annotated the complete chloroplast genome sequence of *M. salicina* for the first time. We analyzed the general features of *M. salicina* and constructed a phylogenetic tree based on 15 Lauraceae species. This study will contribute to species identification and ecological conservation.

Fresh leaves of *M. salicina* were sampled from Panlong District, Kunming City, Yunnan Province, China (24°23′N, 102°10′E). We conducted this research with the consent of the local government and the Kunming Institute of Botany, Chinese Academy of Sciences. The voucher samples and DNA were stored at Qingdao University of Science and Technology (Chao Shi, chsh1111@aliyun.com) under the specimen code MC202114. Fresh leaves tissue with no obvious signs of disease were collected and preserved in silica gel. Complete genomic DNA was extracted from fresh leaves using modified CTAB (Porebski et al. 1997). Both the quantity and quality of the extracted DNA were assessed spectrophotometrically, while the integrity was assessed using 1% (w/v) agarose gel electrophoresis. Illumina TruSeq library preparation kits (Illumina, San Diego, CA, USA) were used to prepare DNA insert libraries of approximately 500 bp paired ends according to the manufacturer's protocol. These libraries were sequenced on the Illumina HiSeq 4000 platform in Novogene (Beijing, China) and generated raw data of 150 bp paired-end reads. About 4.3 Gb high quality, 2 × 150 bp paired-end raw reads were obtained and were used to assemble the complete chloroplast genome of *M. salicina* (Wang et al. 2018). We assembled the chloroplast genome of *M. salicina* using NOVOPlasty v4.3.1 (Dierckxsens et al. 2017) and annotated the chloroplast genome with GeSeq (Tillich et al. 2017). We used Sequin to manually correct codons and gene boundaries.

The chloroplast genome of *M. salicina* (GenBank accession MZ442605) presented a typical quadripartite structure (Wicke et al. 2011) with a total length of 153,943 bp. The large single copy (LSC) region was 93,689 bp in length, and the small single copy (SSC) region was 20,070 bp in length. Two inverted repeat (IR) regions were 20,092 bp in length. A total of 128 genes were found in the chloroplast genome of *M. salicina*, with a GC content of 39.1%. About 128 genes were comprised of 84 protein-coding genes, 36 tRNAs and 8 rRNAs. We performed a phylogenetic analysis of *M. salicina* to reveal its evolutionary relationships with other Lauraceae species. The chloroplast genome sequences of 14 Lauraceae species were obtained from NCBI. *Cinnamomum tenuipile* was used as an outgroup. The chloroplast genomes of the 14 Lauraceae and the newly assembled *M. salicina* were aligned using MAFFT v725 (Katoh and Standley 2013). GTR-GAMMA (GTR + G) model was selected by Modeltest (Posada and Crandall 1998) based on the Bayesian information content (BIC) criterion. The maximum likelihood (ML) method was used for 1000 bootstraps by MEGA-X software (Kumar et al. 2018). The phylogenetic tree showed that *M. salicina* closely was related to *M. yunnanensis* and *Machilus* was closely sister to *Phoebe* (Figure 1). There were parallels between these phenomena and previous studies (Chen et al. 2017; Song et al. 2017).

**Figure 1.**
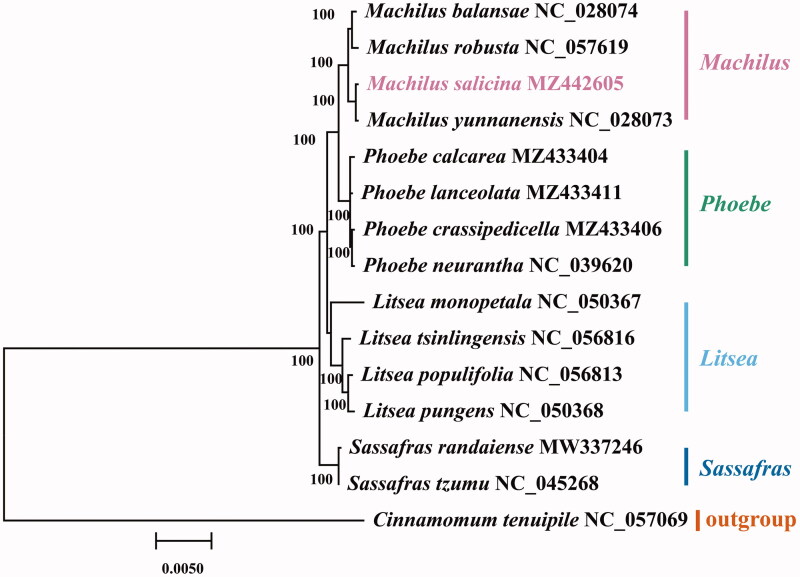
The maximum likelihood tree states the phylogenetic position of *M. salicina* in Lauraceae, with the number on each node denoting the bootstrap support value. The species is followed by the chloroplast genome sequence accession number that was used by GenBank.

## Data Availability

The genome sequence data that support the findings of this study are openly available in GenBank of NCBI at (https://www.ncbi.nlm.nih.gov/) under the accession MZ442605. The associated BioProject, SRA, and Bio-Sample numbers are PRJNA786751, SRR17153751, and SAMN23720018, respectively.
